# Intestinal barrier function is maintained with aging – a comprehensive study in healthy subjects and irritable bowel syndrome patients

**DOI:** 10.1038/s41598-019-57106-2

**Published:** 2020-01-16

**Authors:** Ellen Wilms, Freddy J. Troost, Montserrat Elizalde, Bjorn Winkens, Paul de Vos, Zlatan Mujagic, Daisy M. A. E. Jonkers, Ad A. M. Masclee

**Affiliations:** 1 Division Gastroenterology-Hepatology, Department of Internal Medicine; NUTRIM School of Nutrition and Translational Research in Metabolism, Maastricht University, Maastricht, The Netherlands; 2 Top Institute Food and Nutrition, Wageningen, The Netherlands; 3 Food Innovation and Health Research, Centre for Healthy Eating and Food Innovation, Maastricht University, Venlo, The Netherlands; 4 Department of Methodology and Statistics; CAPHRI, Care and Public Health Research Institute, Maastricht University Medical Center, Maastricht, The Netherlands; 5 Department of Pathology and Medical Biology, section Immunoendocrinology, University of Groningen, University Medical Center Groningen, Groningen, The Netherlands

**Keywords:** Gastroenterology, Risk factors

## Abstract

Animal studies have shown that intestinal barrier function is compromised with aging. We aimed to assess the effects of aging on intestinal barrier function in humans *in vivo* and *ex vivo*. In this cross-sectional study, healthy subjects and subjects with irritable bowel syndrome (IBS) of older (65–75 years) and young adult age (18–40 years) were compared. *In vivo* gastrointestinal site-specific permeability was assessed by a multi-sugar test, taking into account potential confounders. Sigmoid biopsies were collected from subgroups of healthy young adults and elderly for *ex vivo* Ussing chamber experiments, gene transcription of barrier-related genes and staining of junctional proteins. No significant differences between healthy young adults and elderly were found for small intestinal, colonic and whole gut permeability (*P* ≥ 0.142). In IBS patients, gastroduodenal and colonic permeability did not differ significantly (*P* ≥ 0.400), but small intestinal and whole gut permeability were higher in elderly versus young adults (*P* ≤ 0.009), mainly driven by the IBS-diarrhea subtype. Ussing chamber experiments with or without stressor (*P* ≥ 0.052), and relative expression of intestinal barrier-related genes (*P* ≥ 0.264) showed no significant differences between healthy elderly and young adults, as confirmed by immunofluorescent stainings. Overall, the functional capacity of the intestinal barrier is maintained in elderly.

## Introduction

Along with the rising life expectancy, the aging population is steadily increasing worldwide. In 2010, 8% of the world population was aged 65 years or older, and this proportion is expected to reach 16% by 2050, leading to substantial increases in direct and indirect health care costs^1^. The associated functional decline of several organs and tissues, including those of the gastrointestinal (GI) tract and the immune system, contributes to higher vulnerability to infections with aging and age-related co-morbidities^2,3^. With respect to GI physiology and function, a recent review by our group showed that the aging process is associated with small, subtle alterations at both the organ and cellular level^4^. Moreover, the GI mucosal immune function has been found to decline with aging^5^. Based on data of mice, rat and baboon studies it has been stated that intestinal barrier function also decreases with aging, as reflected by an increased paracellular intestinal permeability^6–9^. Intestinal permeability is an important functional feature of the intestinal epithelial barrier^10^. Increased intestinal permeability may lead to permeation of noxious luminal substances into the intestinal mucosa, inducing local and systemic immune activation, and may contribute to *e.g*. an increased infection risk, inflammation, and GI symptoms. So far, a few human studies have investigated the effects of aging on small intestinal or colonic barrier function. In these studies, most of which addressed the small intestine, no differences in sugar excretion ratios between age groups were found^11–14^. However, the impact of potential confounders such as medication use (*e.g*. proton pump inhibitors (PPIs) and non-steroidal anti-inflammatory drugs (NSAIDs)) have not been considered in previous studies, while these drugs are widely used by elderly and less in younger adults. Futhermore, most studies on aging focused on healthy subjects, while it may be also valuable to get insight in barrier function in subjects with mild disturbances in GI health, such as irritable bowel syndrome (IBS) patients, in whom intestinal barrier disfunction has been shown previously^15^, but has not been studies with regard to age. Paracellular permeability is regulated by intercellular junctional complexes^16^. At present, data from human studies with regard to aging on the expression and function of tight junction proteins, sealing the epithelial barrier, are not available.

In summary, available data on the effects of aging on the intestinal barrier in humans are very limited and when present, often conflicting^17^. Therefore, a comprehensive study on the effects of aging on intestinal barrier function is needed. Our aim was to study the effects of aging on human intestinal barrier function in combined *in vivo* and *ex vivo* experiments, by determining GI permeability and the expression of barrier related genes. We hypothesized that intestinal permeability is increased and the expression of intestinal barrier related genes is lower in elderly compared with young adults, in both healthy subjects and IBS patients.

## Results

### Intestinal permeability *in vivo*

Assessment of intestinal permeability *in vivo* by the multi-sugar test was performed in 100 healthy individuals including 48 elderly and 52 young adults, as well as in 48 IBS patients including 21 elderly and 27 young adults. Subject characteristics are shown in Table 1. BMI (*P* < 0.001) and PPI use (*P* = 0.017) were significantly higher in healthy elderly compared with healthy young adults.

**Table 1:** Subject characteristics per age group of the healthy individuals and IBS patients undergoing the multi-sugar test for *in vivo* assessment of the intestinal permeability.

	Healthy individuals		*P-*value	IBS patients		*P*-value
	Young adults (n = 52)	Elderly (n = 48)		Young adults (n = 27)	Elderly (n = 21)	
Age (yrs, mean ± SD)	23.1 ± 4.3	69.7 ± 2.8	<0.001	29.4 ± 6.5	71.1 ± 4.0	<0.001
Female (%)	57.7	43.8	0.164	59.3	66.7	0.599
BMI (kg/m^2^, mean ± SD)	22.9 ± 2.7	25.8 ± 2.7	<0.001	25.4 ± 5.2	25.5 ± 3.2^#^	0.896
Medication (%)						
PPI	0	10.4	0.017	26.9^#^	14.3	0.293
NSAID	N.A	N.A.	N.A.	7.7^#^	23.8	0.123
IBS subtype (%)						
IBS-C				18.5	28.6	0.412
IBS-D	N.A.	N.A.	N.A.	40.7	38.1	0.849
IBS-M				37.0	28.6	0.535
IBS-U				3.7	4.8	0.857

BMI: body mass index, IBS: irritable bowel syndrome, IBS-C: constipation-predominant irritable bowel syndrome, IBS-D: diarrhea-predominant irritable bowel syndrome, IBS-M: irritable bowel syndrome characterized by a mixed pattern, IBS-U: Unsubtyped irritable bowel syndrome, N.A: not applicable, NSAID: nonsteroidal anti-inflammatory drugs, PPI: proton-pump inhibitors. Age and BMI were compared between age groups with the use of an independent samples t-test. Sex, medication and IBS subtype were compared between age groups with the use of a Pearson Chi-square test. ^#^One missing value for this variable.

**Figure 1:**
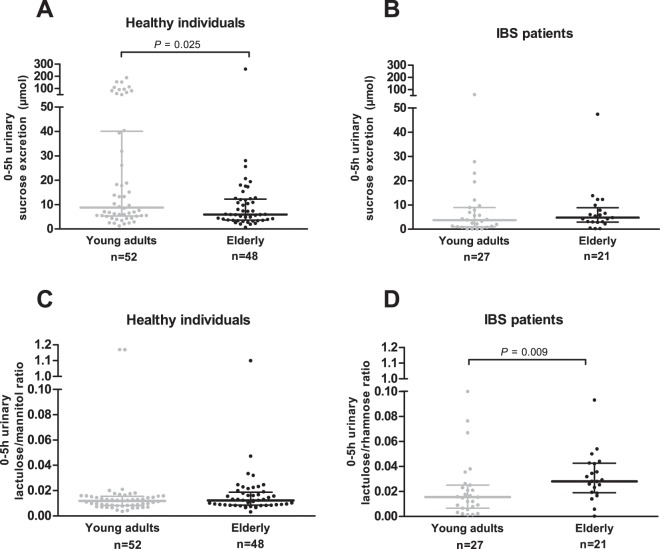
Gastroduodenal and small intestinal permeability *in vivo* comparing young adults vs. elderly. (**A**) 0–5 h urinary sucrose excretion in healthy individuals. (**B**) 0–5 h urinary sucrose excretion in IBS patients. (**C**) 0–5 h urinary lactulose/mannitol ratio in healthy individuals. (**D**) 0–5 h urinary lactulose/rhamnose ratio in IBS patients. Values are presented in scatter plots with median line and IQR (25–75th interquartile range). Urinary sugar excretions and ratios were compared between age groups with the use of Mann-Whitney U-tests.

Gastroduodenal permeability as assessed by the 0–5 h urinary sucrose excretion was lower in healthy elderly compared with young healthy adults (*P* = 0.025), but did not differ significantly between elderly and young IBS patients (*P* = 0.400) (Fig. 1A,B, respectively). The 0–5 h urinary L/M ratio reflecting small intestinal permeability in healthy subjects was not significantly different between elderly and young adults (*P* = 0.214) (Fig. 1C), while in the IBS group, small intestinal permeability as determined by the 0–5 h urinary L/R ratio was higher in elderly compared with young adults (*P* = 0.009) (Fig. 1D).

**Figure 2:**
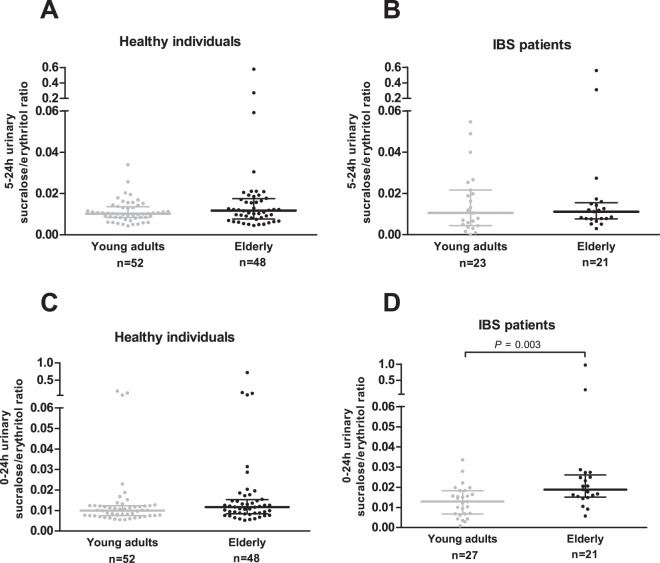
Colonic and whole gut permeability *in vivo* comparing young adults vs. elderly. (**A**) 5–24 urinary sucralose/erythritol ratio in healthy individuals. (**B**) 5–24 urinary sucralose/erythritol ratio in IBS patients. (**C**) 0–24 urinary sucralose/erythritol ratio in healthy individuals. (**D**) 0–24 urinary sucralose/erythritol ratio in IBS patients. Values are presented in scatter plots with median line and IQR (25–75th interquartile range). Urinary sugar ratios were compared between age groups with the use of Mann-Whitney U-tests.

Colonic permeability *in vivo* as determined by the 5–24 h urinary S/E ratio was not significantly different between healthy elderly and healthy young adults (*P* = 0.227), nor between elderly and young IBS patients (*P* = 0.664) (Fig. 2A,B, respectively). The 0–24 h urinary S/E ratio, as measure for whole gut permeability *in vivo*, is not significantly different between healthy elderly and healthy young adults (*P* = 0.061), whereas in IBS patients, the 0–24 h urinary S/E ratio was higher in elderly compared with young adults (*P* = 0.003) (Fig. 2C,D, respectively).

As demographics and medication use may influence intestinal permeability *in vivo*, their impact was accounted for by multivariable linear regression analysis as shown in Supplementary Tables S2 (healthy individuals) and S3 (IBS patients). In healthy individuals, the 0–5 h urinary sucrose excretion (B = −16.430 (95% CI −35.855; 2.996)), 0–5 h urinary L/M ratio (B = −0.051 (95% CI −0.140; 0.038)) and 0–24 h urinary S/E ratio (B = −0.025 (95% CI −0.011; 0.060)) were not significantly affected by age group (elderly vs. young adults). However, healthy elderly had on average a significantly higher 5–24 h urinary S/E ratio (B = 0.030 (95% CI 0.001; 0.058)) compared with healthy young adults. In IBS patients, the 0–5 h urinary sucrose excretion (B = 1.391 (95% CI −5.549; 8.331)) as well as 0–5 h urinary L/R ratio (B = 0.007 (95% CI −0.006; 0.020)), 5–24 h urinary S/E ratio (B = 0.040 (95% CI −0.021; 0.101)) and 0–24 h urinary S/E ratio (B = 0.066 (95% CI −0.021; 0.153)) were not significantly influenced by age group (elderly vs. young adults).

Sex, BMI and PPI use did not significantly affect urinary sugar excretions and ratios in the healthy subjects (Supplementary Table S2). In IBS patients, the 0–5 h urinary sucrose excretion, 5–24 h urinary S/E ratio and 0–24 h urinary S/E ratio were also not significantly influenced by PPI use, NSAID use or IBS subtype (Supplementary Table S3). However, diarrhea-predominant irritable bowel syndrome (IBS-D) patients had on average a significantly higher 0–5 h urinary L/R ratio (B = 0.018 (95% CI 0.004; 0.031)) compared with other subtypes.

### Intestinal permeability *ex vivo*

**Figure 3:**
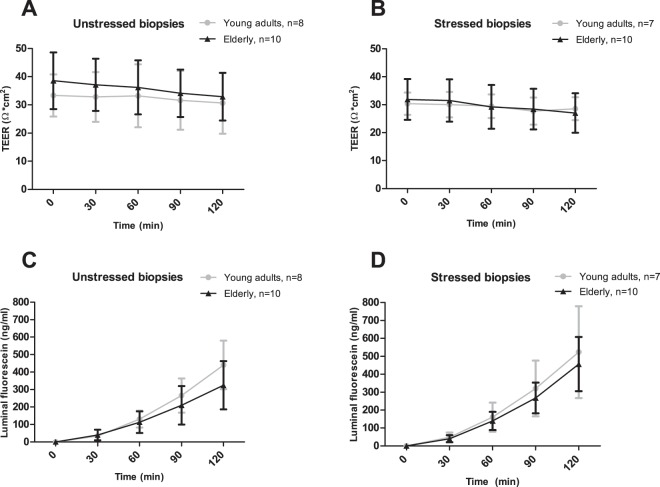
Intestinal permeability *ex vivo* comparing healthy young adults vs. healthy elderly by mounting fresh sigmoid colon biopsies in an Ussing chamber system, and assessing transepithelial electrical resistance (TEER) and luminal fluorescein concentration at t = 0, t = 30, t = 60, t = 90, t = 120 min. (**A**) TEER in unstressed biopsies. (**B**) TEER in biopsies stressed by 1 µg/ml Compound 48/80 at t = 0. (**C**) Luminal fluorescein concentration in unstressed biopsies. (**D**) Luminal fluorescein concentration in biopsies stressed by 1 µg/ml Compound 48/80 at t = 0. In the young adult group, two subjects were removed from the unstressed biopsy analyses, and three subjects were removed from the stressed biopsy analyses because baseline values were not meeting the quality criteria for viability. Means and standard deviations are visualized. TEER and luminal fluorescein were compared between age groups using random intercept linear mixed model analyses including age group, time and age group x time as fixed factors and correction for t = 0 values. *P*-values per time point were corrected for multiple testing by calculating the false-discovery-rate (FDR) of Benjamini-Hochberg.

Ussing chamber experiments were performed to study TEER and luminal fluorescein concentration as functional indicators of paracellular permeability, in unstressed and stressed biopsies of 10 healthy elderly (mean ± SD: 70.7 ± 2.8 yrs) and 10 healthy young adults (24.0 ± 5.4 yrs). Mean BMI was found to be significantly higher in elderly (27.3 ± 1.7 kg/m^2^) compared with young adults (23.4 ± 3.2 kg/m^2^; *P* = 0.005). Sex, PPI use and NSAID use did not differ significantly between elderly (20% female, 20% PPI use, 0% NSAID use) and young adults (30.0% female, 0% PPI use, 0% NSAID use; all *P* ≥ 0.136). After FDR correction for multiple time points, TEER did not significantly differ between elderly and young adults in unstressed (all *P* ≥ 0.208), nor in stressed biopsies (all *P* ≥ 0.096) (Fig. 3A,B, respectively). In both unstressed biopsies (all *P* ≥ 0.052) and stressed biopsies (all *P* ≥ 0.760), luminal fluorescein concentration did not differ significantly between elderly and young adults (Fig. 3C,D, respectively).

### Gene transcription of barrier related genes

Relative expression of junctional complex (*e.g*. tight junctions and adherens junctions), defense and immune-related (*e.g*. human defensins, cytokines and toll-like receptor) genes in sigmoid biopsies were studied because these are directly or indirectly related to paracellular permeability and barrier function in general. Before correcting for multiple testing, only cadherin 1 (*P* = 0.047) was higher and toll-like receptor 1 (*P* = 0.024) was lower in healthy elderly compared with healthy young adults. After correcting for multiple testing, there were no significant differences (all *P* ≥ 0.264) between healthy elderly and healthy young adults in the relative expression of junctional complex, defense and immune-related genes (Table 2).

**Table 2:** Relative expression of junctional complex (*e.g*. tight junction related and adherens junctions), defense and immune related (*e.g*. human defensins, cytokines and toll-like receptor) genes in sigmoid biopsies of young adults and elderly.

Cluster	Gene name	Young adults			Elderly			*P-*value	Benjamini Hochberg *P*-value
		N	Mean	SD	N	Mean	SD		
Junctional complex related genes	TJP1 (ZO-1)	10	1.13	0.03	10	1.14	0.02	0.641	0.673
	OCLN	10	1.18	0.02	10	1.19	0.02	0.314	0.673
	CLDN2	5	1.36	0.03	6	1.32	0.07	0.269	0.673
	CLDN3	10	1.16	0.02	10	1.17	0.02	0.556	0.673
	CLDN4	10	1.10	0.02	10	1.11	0.02	0.673	0.673
	MLCK	10	1.15	0.03	10	1.15	0.03	0.534	0.673
	CDH1	10	1.15	0.01	10	1.17	0.02	0.047	0.376
	CTNNB1	10	1.12	0.01	10	1.13	0.02	0.386	0.673
Defense and immune related genes	CAMP	9	1.30	0.05	10	1.28	0.06	0.462	0.726
	DEFB1	10	1.15	0.03	10	1.16	0.03	0.951	0.951
	MUC2	10	1.01	0.03	10	1.01	0.02	0.559	0.769
	TFF3	10	0.98	0.04	10	0.98	0.04	0.898	0.951
	IL1B	10	1.35	0.05	9	1.32	0.04	0.233	0.667
	IL10	9	1.25	0.03	9	1.23	0.06	0.300	0.667
	TNF	9	1.35	0.06	5	1.34	0.04	0.805	0.951
	TLR1	10	1.18	0.04	10	1.13	0.04	0.024	0.264
	TLR2	9	1.26	0.05	10	1.23	0.06	0.354	0.667
	TLR4	10	1.21	0.03	10	1.19	0.03	0.063	0.347
	TLR6	8	1.29	0.04	7	1.27	0.06	0.364	0.667

TJP1 (ZO-1): Tight junction protein 1 (*i.e*. Zona Occludens-1), OCLN: Occludin, CLDN: Claudin, MLCK: Myosin light chain kinase, CDH1: Cadherin 1, CTNNB1: Catenin beta 1, CAMP: Cathelicidin antimicrobial peptide, DEFB1: Defensin beta 1, MUC2: Mucin 2, TFF3: Trefoil factor 3, IL: Interleukin,, TNF: Tumor necrosis factor, TLR: Toll like receptor. GAPDH was used as reference gene. Values are presented as mean ± SD. Differences between age groups were tested by independent-samples T Tests. *P-*values were corrected for multiple testing by calculating the false-discovery-rate of Benjamini Hochberg per cluster.

### Immunofluorescent stainings of TJP1 (ZO-1) and occludin

**Figure 4:**
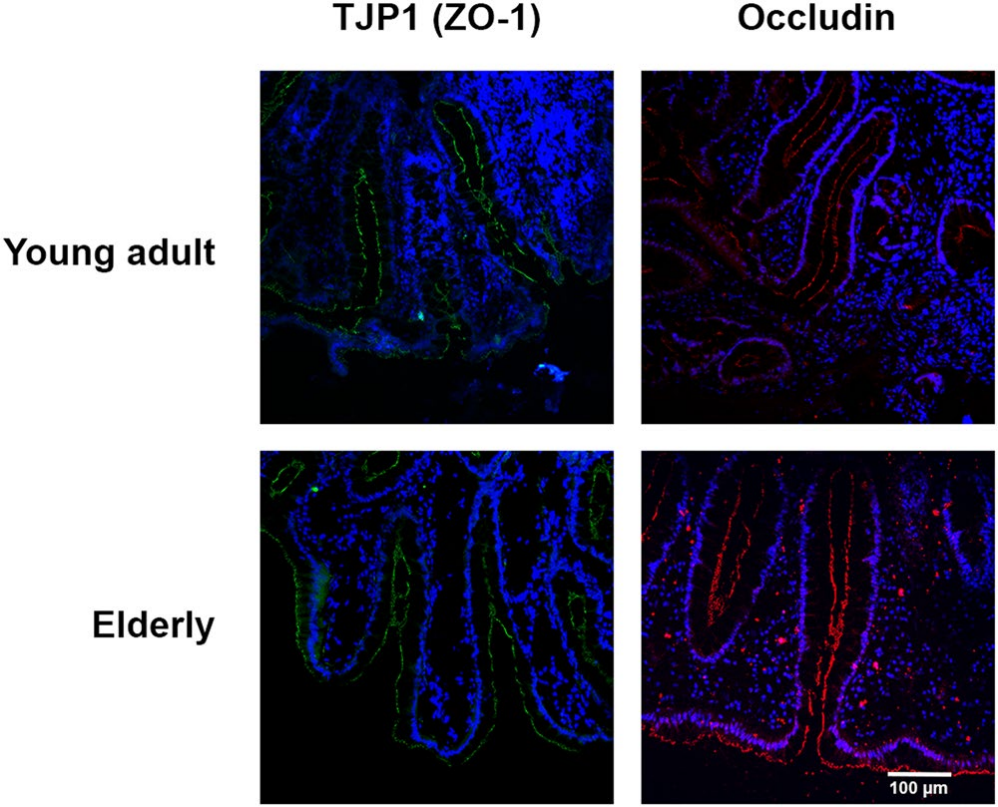
Representative images of tight junction proteins TJP1 (ZO-1) (green) and occludin (red) immunofluorescent stainings in sigmoid biopsy sections showing glandular epithelium of a healthy young adult and healthy elderly. Scale bar represents 100 µm. Blue counterstaining (DAPI) shows nuclei. TJP1: Tight junction protein 1.

Representative images of immunofluorescent staining TJP1 (ZO-1) and occludin in sigmoid biopsy sections of a healthy elderly and healthy young adult are presented in Fig. 4. TJP1 (ZO-1) and occludin showed continuous staining without disruption along the villous epithelium. No apparent differences were noted between the elderly and young adults. These observations are in line with quantitative analyses of TJP1 (ZO-1) and occludin gene transcription levels as reported in Table 2.

## Discussion

In this study, we evaluated various, different and complementary aspects of GI barrier function to assessed the effects of aging in humans. Overall, GI segment-specific permeability and expression of barrier-related genes were not significantly affected by aging in healthy individuals. In IBS, a condition which is generally considered to be associated with mild alterations in gut function, small intestinal permeability was higher in the elderly. This difference was partly explained by the IBS-D subtype. We therefore rejected the study hypothesis that intestinal barrier function and the expression of barrier related genes is altered with aging per se in healthy subjects and in mild GI disorders.

Up to now, previous studies on aging and intestinal permeability *in vivo* have been critically debated because of methodological issues. One study^11^ included a heterogeneous group of patients and hospital staff. In two other studies^12,13^ high dosages of mannitol (5 g) and lactulose (10 g) were used, which is in this dosage considered a laxative, and therefore probably inducing osmotic effects^18^. We applied the validated multi-sugar test, using low sugar dosages (0.5–1 g per sugar probe), to determine site-specific paracellular intestinal permeability *in vivo* in well defined groups of healthy individuals and IBS patients, taking into account factors potentially influencing these parameters. Gastroduodenal permeability was determined by assessing urinary sucrose excretion in the 0–5 h fasting period of the multi-sugar test. Urinary sucrose excretions were significantly lower in the healthy elderly compared with healthy young adults. Although we cannot exclude that this may in part be due to more outliers in the young adults group than in the elderly, age group was not significant in the multivariable linear regression analysis by correcting for potential confounders. Therefore our findings are in line with previous findings in healthy adults^19,20^ and indicating the initial difference in gastroduodenal permeability should be interpreted with caution. Moreover, in elderly and young adult IBS patients we observed a comparable gastroduodenal permeability. To the best of our knowledge, this is the first study investigating gastroduodenal permeability and aging. A clear reason for the outliers in the healthy young adults is lacking, but it should be acknowledged that urinary sucrose excretion was not corrected for potential differences in for example transit time or renal clearance as no transcellar probe was available to correct for. Although some debates are ongoing on the optimal timing of urine collection, in the current study the 0–5 urinary L/R ratio and L/M ratio used in the current study are considered to reflect small intestinal permeability in healthy and IBS individuals, respectively. As medians of 0–5 h urinary L/R and L/M ratios were in the same range, the impact of using different disaccharides as a transcellar probe indeed seemed negligible. We showed that small intestinal permeability did not differ significantly between healthy elderly and healthy young adults, which is in line with previous observations in healthy individuals^12,13^. In IBS patients however, small intestinal permeability was higher in elderly compared with young adults. Furthermore, we showed that the IBS-D subtype was associated with an increased small intestinal permeability in these elderly, confirming previous observations of the Maastricht IBS cohort^15^ and others^21,22^. This indicates that the observed increase was not due to the aging per se. Using univariate non-parametric comparisons (*i.e*. Mann-Whitney U-tests), no significant differences were found in colonic permeability as measured by the 5–24 h urinary S/E ratio between elderly and young adults neither in healthy individuals, nor in IBS patients. These findings are largely in line with the comparable 5–24 h urinary S/E ratios between elderly without GI symptoms and younger healthy individuals and as shown by Ganda Mall *et al*.^14^. However, parametric, multivariable linear regression analyses showed that healthy elderly had on average a significantly higher 5–24 h urinary S/E ratio compared with healthy young adults. This was caused by a higher sucralose flux (data not shown), pointing towards a slightly increased paracellular permeability. The colon harbors a complex dense environment of not only beneficial microbes, but also potentially harmful microbes and antigens. Maintaining a well-functioning colonic barrier function with aging is advantageous since it will limit permeation of such components into the intestinal mucosa, and prevent mucosal damage, local and systemic immune activation. The 0–24 h urinary S/E ratio, reflecting whole gut permeability, did not differ significantly between elderly and healthy young adults, but in IBS patients a higher whole gut permeability was observed in elderly compared with young adults. This can at least partly be explained by the observed effect of aging on small intestinal permeability in the diarrhea-predominant IBS patients. In the current study, sugar ratios were used to correct for *e.g*. transit time and renal clearance. Additionally, to determine intestinal permeability *ex vivo* as well as the susceptibility to a stressed condition, Ussing chamber experiments with sigmoid biopsies of a subgroup of healthy individuals were conducted. We demonstrated that after FDR correction, TEER and luminal fluorescein concentrations were not significantly different between elderly and young adults. To our knowledge, one other study compared sigmoid biopsies of elderly with GI symptoms versus young healthy controls, and found no significant difference in TEER, although fluorescein flux and horseradish peroxidase (HRP) were significantly higher in elderly with GI symptoms compared with healthy controls^14^. Unfortunately, no biopsies of IBS patients were available for Ussing chamber experiments. Moreover, a human study by Man *et al*. performed Ussing chamber experiments in unstressed ileum biopsies and found that TEER values were significantly lower in healthy elderly (67–77 yrs) compared with healthy adults (20–40 yrs), while HRP flux remained unchanged^23^. TEER does also reflect transcellular ion transport and thereby does not necessarily indicated alterations in the junctional complex. Differences between the locations of tissue sampling between the previously reported observations and the current study, the ileum and the colon, respectively, further impede direct comparison of the results. We used sigmoid biopsies to limit the invasiveness for the subjects and as a tightly controlled permeability is considered especially important in the colon with its high bacterial load. We did apply a mild physiological relevant stressor (1 µg/ml Compound 48/80), which resulted in an average increase in luminal fluorescein concentrations of 28.7% and average decrease in TEER of 13.2% after 120 minutes (data not shown) to check for a potential increased susceptility to stressed conditions. In the by Compound 48/80 mildly stressed biopsies, no differences were observed between young adults and elderly in in TEER and luminal fluorescein concentrations. This point towards a maintained adaptive capacity of the tissue samples with aging.

Relative expression levels of genes related to the junctional complex, innate and adaptive immunity and barrier function in general, as analyzed by qPCR, showed no significant differences between healthy adults and healthy elderly after FDR correction. These observations were supported by immunofluorescent stainings of TJP1 (ZO-1) and occludin in representative colon biopsy sections, confirming that the junctional complex seems to remain intact with aging. In the study by Man *et al*., claudin-2 and IL-6 expression levels were found to be higher in ileum biopsies of elderly (67–77 yrs) compared with young adults (20–40 yrs), but no changes were found in TJP1 (ZO-1), occludin, JAMA-1, IFN-γ, IL-1β and TNF-α^23^. In that study, while testing a relativly large number of genes, the results were not corrected for multiple testing and therefore should be interpreted with care. In the current study corrections for multiple testing were applied and we show no statistically signicant differences between age groups. Secondly, findings of the ileum and sigmoid colon are difficult to compare because junctional complexes are stronger in the colon compared with small intestine. Lastly, the intestinal barrier is not static, but constantly remodeling in order to selectively regulate intestinal permeability. For example, TJP1 (ZO-1) stabilizes claudin strands and tightens them to the actin cytoskeleton. Therefore, single gene expression needs to be interpreted with care. In the current study, we investigated a broad range of junctional complex-related genes showing no significant differences with aging. The intestinal barrier is not only formed by the epithelial layer, but includes overlying mucus and produces for example antimicrobial proteins. Therefore we analyzed innate defense and immune related genes, but these did not differ significantly between healthy adults and elderly. Overall, our findings in *ex vivo* experiments are in line with the measured *in vivo* intestinal permeability.

The strength of the current study is that the effects of aging on gastroduodenal, small intestinal and colonic permeability were investigated in distinct age groups of healthy individuals and IBS patients using a combined *in vivo* and *ex vivo* approach, enabling to study functional as well as structural aspects of intestinal barrier function. This study showed that intercellular junctional functionality regulating paracelular permeation, was maintained with aging in both healthy individuals and IBS patients. Only small intestinal permeability was increased in IBS-D, independent of age. Moreover, the expression of barrier related genes was comparable between healthy elderly and healthy young adults. Intact intestinal permeability regulation can prevent permeation of noxious luminal substances into the intestinal mucosa, with subsequent local and systemic immune activation. In conclusion, although age-related factors such as medication use and co-morbidities may impact barrier function, we did not find an indication for impaired intestinal permeability in aging per se.

## Methods

### Study design and participants

**Figure 5:**
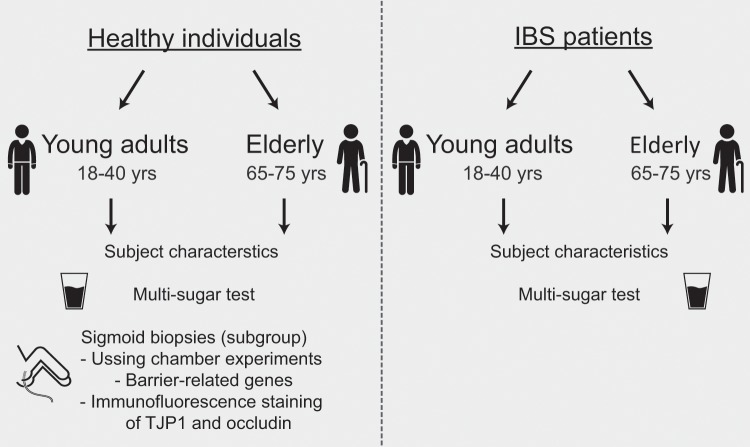
Overview of the study population and measurements.

In this cross-sectional study, baseline data of healthy individuals who participated in a human intervention study, and of IBS patients of the Maastricht IBS cohort^15^ were used. Both studies were approved by the Medical Ethics Committee of the University Hospital Maastricht and Maastricht University, were registered in the US National Library of Medicine (http://www.clinicaltrials.gov, NCT02376270 and NCT00775060, respectively), and performed between November 2009 and April 2016, in accordance with the Declaration of Helsinki (latest amendment in Fortalesa, Brasil, 2013) and Dutch Regulations on Medical Research involving Human Subjects (1998). Healthy individuals were recruited by public advertisements and key exclusion criteria were GI diseases, abdominal surgery interfering with GI function, pregnancy, and use of medication influencing intestinal permeability such as NSAIDs. IBS patients were recruited via the Maastricht University Medical Center + Gastroenterology-Hepatology outpatient clinic and via regional general practices, and were diagnosed and classified by the Rome III criteria with exclusion of organic diseases when indicated, as described previously^15^. To assess the effects of aging, two groups were included in both the healthy and the IBS populations: *i.e*. elderly 65–75 years and young adults 18–40 years to create distinct age groups (Fig. 5). All participants gave written informed consent before participation.

### Outcome parameters

In the current study, various, different and complementary measurements of barrier function were measured (Fig. 5). All healthy and IBS individuals were subjected to a standardized and validated multi-sugar urinary recovery test to assess GI segment-specific intestinal permeability *in vivo*. From a subgroup of the healthy subjects (10 elderly and 10 young adults) colonic biopsies were collected for *ex vivo* analyses of intestinal permeability by Ussing chamber experiments as well as for gene and protein analyses of intestinal barrier related proteins. These subjects underwent flexible sigmoidoscopy without bowel preparation. Biopsy specimens were taken with a jumbo biopsy forceps (Boston Scientific, Kerkrade, the Netherlands) from a standardized location, *i.e*. 30 cm proximal to the anus. Six tissue samples were immediately transported to the laboratory for Ussing chamber experiments. One tissue sample was snap frozen in liquid nitrogen and stored at −80 °C for gene transcription analyses. One tissue sample was mounted in Tissue-Tek® optimal cutting temperature compound (Sakura, Finetek, Tokio, Japan), snap frozen in liquid nitrogen and stored at −80 °C for immunofluorescent staining of Tight junction protein 1 (TJP1; *i.e*. Zona Occludens-1) and occludin.

### Multi-sugar test for analysis of intestinal permeability *in vivo*

Segment-specific permeability of the GI tract was assessed with a validated multi-sugar test^18,24^. On the day prior to, as well as during the test, subjects were asked to refrain from excessive physical exercise and consumption of alcohol. After fasting overnight, a mix of water-soluble sugar probes were ingested. IBS patients ingested 1 g sucrose (Van Gilse, Dinteloord, the Netherlands), 1 g lactulose (Centrafarm Services, Etten-Leur, the Netherlands), 0.5 g L-rhamnose (Danisco, Copenhagen, Denmark), 1 g sucralose (Tate and Lyle Ingredients Americas, Decatur, IL, USA) and 1 g erythritol (Now Foods, Bloomindale, IL, USA), dissolved in 200 ml tap water. Healthy individuals ingested the same mixture, except for 0.5 g mannitol (Roquette, Lestrem, France) instead of L-rhamnose. It was not possible to use L-rhamnose as permeation marker in that study, as the subsequent intervention from which these baseline data were obtained entailed supplementation with pectin, a non digestible carbohydrate that contains rhamnose residues. These residues would have interfered with a multisugar urinary recovery test containing L-rhamnose. Mannitol and L-rhamnose are both disaccharides with the same intestinal permeation characteristics, therefore we do not expect this to hinder comparability of the data. After ingestion, all participants collected 24 hours (h) urine in two separate fractions; 0–5 h and 5–24 h. During the first 5 h of urine collection, participants were asked to refrain from any food or drinks, except for water ad libitum. Thereafter, participants were allowed to eat and drink as preferred, except for sucralose containing foods. After the collection periods, volumes of urine fractions were determined and aliquots were frozen at −80 °C until analysis. Sugar probes were analyzed by isocratic ion-exchange High Performance Liquid Chromatography with mass spectrometry as described previously^18,24^. Gastroduodenal permeability was determined by sucrose excretion in 0–5 h urine, small intestinal permeability by 0–5 h urine lactulose to mannitol (L/M) ratio in healthy individuals and lactulose to rhamnose (L/R) ratio in IBS patients, colonic permeability by sucralose to erythritol (S/E) ratio in 5–24 h urine, and whole gut permeability by sucralose to erythritol (S/E) ratio in 0–24 h urine.

### *Ex vivo* ussing chamber experiments

Six tissue samples from the sigmoid colon were used for *ex vivo* Ussing chamber experiments as previously described by our group^25^. Three tissue samples were stressed by adding mast cell degranulator Compound 48/80 (1 µg/ml, Sigma-Aldrich, St. Louis, MO, USA) to the serosal compartment. Three non-exposed tissue samples served as controls. At t = 0, 1 mg/ml fluorescein (376 g/mol, Sigma-Aldrich, St. Louis, MO, USA) was added to the serosal compartment. Potential difference (PD), Transepithelial electrical resistance (TEER) and luminal fluorescein concentrations were determined at time point t = 0, 30, 60, 80 and 120 min, respectively. TEER and PD were used as quality criteria for viability. Only samples with a baseline TEER above 20 Ω/cm^2^, or those with baseline TEER between 15–20 Ω/cm^2^ and PD below 0.5 mV, were included for analyses. Lower TEER values and higher fluorescein concentrations are indicators of impaired intestinal permeability.

### Gene transcription of barrier-related genes

Transcription of junctional complex related genes as well as defense and immune related genes associated with barrier function or modulation thereof, were determined in colonic tissue samples. Nucleic acid extraction and purification, RNA isolation and reverse transcription were performed as previously described^26^. Depending on the gene of interest, cDNA was diluted to final concentrations of 20 ng/µl, 40 ng/µl or 80 ng/µl (Supplementary Table S1). Quantitative real-time polymerase chain reaction (qPCR) was performed as described previously^27^. Expressions of target genes were normalized to glyceraldehyde-3-phosphate dehydrogenase (GAPDH) as reference gene.

### Immunofluorescent staining of TJP1 (ZO-1) and occludin

Sigmoid biopsy sections (10 µm) were used for immunofluorescent staining of TJP1 (ZO-1) and occludin as previously described by Elamin *et al*.^28^.

### Statistical analyses

This is considered a first human study applying the multi-sugar test in combination with *ex vivo* analyses. We made use of existing data sets to investigate the effects of aging on intestinal permeability. The results of this study will enable future power calculations in this field, especially because of the complementary range of parameters to determine intestinal barrier function.

Missing data was not imputed, but reported upon in the results. We checked for normality of the data (histograms) and subsequently variables were summarized using median and interquartile range (IQR; 25–75th IQR) or means ± standard deviation for numerical variables, and percentages for categorical variables. Mann-Whitney U-tests and independent-samples T Tests were performed for numerical variables and Chi-square tests for categorical variables to test for differences between age groups (elderly versus young adults). Factors potentially influencing intestinal permeability *in vivo* were tested by multivariable linear regression analysis. These regression models included age group, sex, body mass index (BMI), and PPI use for healthy individuals, and age group, PPI use, NSAID use, and IBS subtype for IBS patients. Differences in longitudinal trends in TEER and luminal fluorescein between age groups were assessed by random intercept linear mixed model analyses with age group (elderly and young adult), time (t = 0, t = 30, t = 60, t = 90, t = 120 min) and ‘age group x time’ as fixed factors, and correction for t = 0 values. All statistical analyses were performed using IBM SPSS Statistics for Windows (version 25.0, Armonk, NY, USA: IBM Corp.). *P*-values ≤ 0.05 (two-sided) were considered statistically significant. Ussing chamber experiments and gene transcription *P*-values were corrected for multiple testing by the false-discovery-rate (FDR) of Benjamini-Hochberg.

## Supplementary information

Supplementary information.

## Data Availability

The datasets generated during and/or analysed during the current study are available from the corresponding author on reasonable request.
