# Supplementary material for: Intestinal barrier function is maintained with aging – a comprehensive study in healthy subjects and irritable bowel syndrome patients
Source: Sci Rep. 2020 Jan 16;10:475. doi: 10.1038/s41598-019-57106-2 (PMC6965102; doi:10.1038/s41598-019-57106-2)
Supplement: Supplementary file 1 — Supplementary information. [file 41598_2019_57106_MOESM1_ESM.pdf]

Supplementary Information

Title

Intestinal barrier function is maintained with aging – a comprehensive study in healthy subjects and irritable bowel syndrome patients

Authors

Ellen Wilms[1,2,*], Freddy J. Troost[1,3], Montserrat Elizalde[1], Bjorn Winkens[4], Paul de Vos[2,5], Zlatan Mujagic[1], Daisy M.A.E. Jonkers[1], Ad A.M. Masclee[1]

Institutions

[1] Division Gastroenterology-Hepatology, Department of Internal Medicine; NUTRIM School of Nutrition and Translational Research in Metabolism, Maastricht University, Maastricht, the Netherlands.

[2] Top Institute Food and Nutrition, Wageningen, the Netherlands.

[3] Food Innovation and Health Research, Centre for Healthy Eating and Food Innovation, Maastricht University, Venlo, The Netherlands.

[4] Department of Methodology and Statistics; CAPHRI, Care and Public Health Research Institute, Maastricht University Medical Center, Maastricht, The Netherlands.

[5] Department of Pathology and Medical Biology, section Immunoendocrinology, University of Groningen, University Medical Center Groningen, Groningen, The Netherlands.

Corresponding author

* PO Box 5800, 6202 AZ Maastricht, the Netherlands

E-mail: e.wilms@maastrichtuniversity.nl

Phone: 0031 43 3884295

Supplementary Table S1. Forward and reverse primer sequences and final cDNA concentrations of all target genes, as determined in sigmoid tissue samples

**Name**	**Description**	**Primer sequences**	**cDNA concentration**
CAMP	Cathelicidin antimicrobial peptide	5'-AGGATTGTGACTTCAAGAAGGACG-3'<br>5'-GTTTATTTCTCAGAGCCCAGAAGC-3'	80 ng/µl
CDH1	Cadherin 1	5'-CACCTGGAGAGAGGCCGCGT -3'<br>5'-AACGGAGGCCTGATGGGGCG -3'	20 ng/µl
CLDN2	Claudin 2	5'-AACTACTACGATGCCTACC-3'<br>5'-GAACTCACTCTTGACTTTGG -3'	20 ng/µl
CLDN3	Claudin 3	5'-TTCATCGGCAGCAACATCATC-3'<br>5'-CGCCTGAAGGTCCTGTGG-3'	20 ng/µl
CLDN4	Claudin 4	5'-ACAGACAAGCCTTACTCC-3'<br>5'-GGAAGAACAAAGCAGAG-3'	20 ng/µl
CTNNB1	Catenin beta 1	5'-GTGCTATCTGTCTGCTCTAGTA -3'<br>5'-CTTCCTGTTTAGTTGCAGCATC -3'	20 ng/µl
DEFB1	Defensin beta 1	5'-CTCTGTCAGCTCAGCCTC-3'<br>5'-CTTGCAGCACTTGGCCTTCCC-3'	20 ng/µl
GAPDH	Glyceraldehyde-3-phosphate dehydrogenase	5'-TGCACCACCAACTGCTTAGC-3'<br>5'-GGCATGGACTGTGGTCATGAG-3'	20 ng/µl
IL1B	Interleukin 1 beta	5'-AAACAGATGAAGTGCTCCTTCCAGG-3'<br>5'-TGGAGAACACCACTTGTTGCTCCA-3'	40 ng/µl
IL10	Interleukin 10	5'-TCAGGGTGGCGACTCTAT-3'<br>5'-TGGGCTTCTTTCTAAATCGTTC-3'	80 ng/µl
MUC2	Mucin 2	5'-GTCAACCCTGCCGACACCTG-3'<br>5'-ACTCACACCAGTAGAAAGGACAGC-3'	20 ng/µl
MLCK	Myosin light chain kinase	5-GCCTGACCACGAATATAAGTT-3'<br>5'-GCTCCTTCTCATCATCATCTG-3'	20 ng/µl
OCLN	Occludin	5'-TCAGGGAATATCCACCTATCACTTCAG-3'<br>5'-CATCAGCAGCAGCCATGTACTCTTCAC-3'	20 ng/µl
TFF3	Trefoil factor 3	5'-CTTGCTGTCCTCCAGCTCT-3'<br>5'-CCGGTTGTTGCACTCCTT-3'	20 ng/µl

TJP1 (ZO-1)	Tight junction protein 1	5’-AGGGGCAGTGGTGGTTTTCTGTTCTTTC-3’<br>5’-GCAGAGGTCAAAGTTCAAGGCTCAAGAGG-3’	20 ng/µl
TLR1	Toll like receptor 1	5’-CAGTGTCTGGTACACGCATGGT-3’<br>5’-TTTCAAAAACCGTGTCTGTTAAGAGA-3’	80 ng/µl
TLR2	Toll like receptor 2	5’-GCCAAAGTCTTGATTGATTGG-3’<br>5’-TATACCACAGGCCATGGAAAC-3’	20 ng/µl
TLR4	Toll like receptor 4	5'-CCTGCGTGAGACCAGAAAGC-3'<br>5'-TCAGCTCCATGCATTGATAAGTAATA-3'	80 ng/µl
TLR6	Toll like receptor 6	5’-GAAGAAGAACAACCCTTTAGGATAGC-3’<br>5’-AGGCAAACAAAATGGAAGCTT-3’	20 ng/µl
TNF	Tumor Necrosis Factor	5'-CCGAGTGACAAGCCTGTAGC-3'<br>5'-GAGGACCTGGGAGTAGATGAG-3'	40 ng/µl

Supplementary Table S2. Multivariable regression analyses of intestinal permeability *in vivo* including 0-5h urinary sucrose excretion, 0-5h urinary L/M ratio, 5-24h urinary S/E ratio and 0-24h urinary S/E ratio in healthy individuals (n=100)

Intestinal permeability parameter	Variable	Adjusted effects B	95% CI	*P*-value
0-5h urinary sucrose excretion	Age group (elderly vs. young adults)	-16.430	-35.855; 2.996	0.096
	Sex (female vs. male)	0.952	-16.560; 18.464	0.914
	BMI	-0.373	-3.631; 2.884	0.821
	PPI use (yes vs. no)	-4.334	-44.018; 35.351	0.829
0-5h urinary lactulose/mannitol ratio	Age group (elderly vs. young adults)	-0.051	-0.140; 0.038	0.257
	Sex (female vs. male)	-0.060	-0.140; 0.020	0.140
	BMI	0.010	-0.005; 0.025	0.204
	PPI use (yes vs. no)	-0.045	-0.227; 0.137	0.623
5-24h urinary sucralose/erythritol ratio	Age group (elderly vs. young adults)	0.030	0.001; 0.058	**0.040**
	Sex (female vs. male)	0.013	-0.012; 0.039	0.301
	BMI	-0.002	-0.007; 0.002	0.323
	PPI use (yes vs. no)	-0.019	-0.077; 0.039	0.521
0-24h urinary sucralose/erythritol ratio	Age group (elderly vs. young adults)	0.025	-0.011; 0.060	0.168
	Sex (female vs. male)	0.003	-0.029; 0.035	0.853
	BMI	-0.002	-0.008; 0.004	0.540
	PPI use (yes vs. no)	-0.025	-0.097; 0.047	0.495

BMI: body mass index, PPI: proton pump inhibitors, B: unstandardized regression coefficient, 95% CI: 95% confidence interval. The multivariable models include age group, sex, BMI and PPI use as independent variables. Assumptions for linear regression were met since there were no influential outliers based on Cooks distance ≤ 0.883, and collinearity was met as indicated by variance inflation factor values ≤ 1.418. R squares of the models were 0.048 for 0-5h urinary sucrose excretion, 0.055 for 0-5h urinary lactulose/mannitol ratio, 0.057 for 5-24h urinary sucralose/erythritol ratio and 0.023 for 0-24h urinary sucralose/erythritol ratio.

Supplementary Table S3. Multivariable regression analyses of intestinal permeability *in vivo* including 0-5h urinary sucrose excretion, 0-5h urinary L/R ratio, 5-24h urinary S/E ratio and 0-24h urinary S/E ratio in IBS patients (n=47)

Intestinal permeability parameter	Variable	Adjusted effects B	95% CI	*P*-value
0-5h urinary sucrose excretion	Age group (elderly vs. young adults)	1.391	-5.549; 8.331	0.688
	PPI use (yes vs. no)	1.593	-6.641; 9.827	0.698
	NSAID use (yes vs. no)	-6.345	-16.568; 3.878	0.217
	IBS subtype (IBS-D vs. other subtypes)	-3.170	-10.357; 4.017	0.378
0-5h urinary lactulose/rhamnose ratio	Age group (elderly vs. young adults)	0.007	-0.006; 0.020	0.316
	PPI use (yes vs. no)	-0.010	-0.025; 0.006	0.216
	NSAID use (yes vs. no)	0.015	-0.004; 0.034	0.126
	IBS subtype (IBS-D vs. other subtypes)	0.018	0.004; 0.031	**0.010**
5-24h urinary sucralose/erythritol ratio [#]	Age group (elderly vs. young adults)	0.040	-0.021; 0.101	0.188
	PPI use (yes vs. no)	-0.006	-0.077; 0.065	0.871
	NSAID use (yes vs. no)	-0.051	-0.138; 0.035	0.239
	IBS subtype (IBS-D vs. other subtypes)	-0.045	-0.109; 0.018	0.157
0-24h urinary sucralose/erythritol ratio	Age group (elderly vs. young adults)	0.066	-0.021; 0.153	0.134
	PPI use (yes vs. no)	-0.014	-0.118; 0.089	0.779
	NSAID use (yes vs. no)	-0.067	-0.196; 0.061	0.297
	IBS subtype (IBS-D vs. other subtypes)	-0.054	-0.144; 0.037	0.237

NSAID: non-steroidal anti-inflammatory drugs, IBS: irritable bowel syndrome, PPI: proton pump inhibitors, B: unstandardized regression coefficient, 95% CI: 95% confidence interval. The multivariable models include age group, PPI use, NSAID use and IBS subtype use as independent variables. Assumptions for linear regression were met since there were no influential outliers based on Cooks distance ≤ 0.736, and collinearity was met as indicated by variance inflation factor values ≤ 1.211. R squares of the models were 0.043 for 0-5h urinary sucrose excretion, 0.210 for 0-5h urinary lactulose/rhamnose ratio, 0.099 for 5-24h urinary sucralose/erythritol ratio and 0.089 for 0-24h urinary sucralose/erythritol ratio. [#] Four missing values for this variable.
